# Strained encounters of the anthropogene: preservation and extinction in Tatsuaki Ishiguro’s ‘It is with the Deepest Sincerity that I Offer Prayers’

**DOI:** 10.1136/medhum-2025-013282

**Published:** 2025-05-30

**Authors:** Lara Choksey

**Affiliations:** 1 English Language and Literature, University College London, London, UK

**Keywords:** biocultures, Genetics, cultural history, narrative ethics, Infectious diseases

## Abstract

Calculating biovalue in an age of planetary emergency involves fraught decisions over what and whom should be saved, over the science used to make these decisions, and the biotechnologies used for this partial salvation. Tatsuaki Ishiguro’s short story, ‘It is with the Deepest Sincerity that I Offer Prayers’, dramatises these decisions through a series of strained encounters in a remote species preservation centre between two molecular biologists and the last two remaining members of a rare species of mouse. Set on Hokkaido, Japan’s northernmost island, largely inhabited by Indigenous Ainu communities until the island’s colonisation in the late 19th century, Ishiguro’s story subverts the teleology of species-life on which conservation models depend. Unsettling what Krithika Srinivasan identifies as human-centred values of well-being (and sexual reproduction) in wildlife conservation, these strained encounters between scientists and test subjects, selectionist methods and theories of life are a genre of what Hannah Landecker has recently called ‘anthropogenic biology’: relations of organic matter shaped by biological control, both affected by and exceeding calculations of scientific efficacy. The article tackles questions of reproductive justice for communities who fall outside core sites of climate mitigation, building on research that shows how conservation projects obscure the realities of environmental pressures caused by industrialisation while upholding a species-centred, genetic ark model of survival. The molecular biologists of Ishiguro’s story encounter something akin to what Landecker calls life as aftermath: ecological flourishing that is historically bound to particular times and places, and which cannot be reproduced indefinitely.

 Tatsuaki Ishiguro’s (2015) short story, ‘It is with the Deepest Sincerity that I Offer Prayers’ from his short story collection, *Biogenesis* (1993), begins with the deaths of two molecular biologists in the early 1990s. Erstwhile laboratory colleagues working in a remote species preservation centre on Hokkaido, Japan’s northernmost island, their deaths are mysterious not in type but in simultaneity: they die within months of each other. More puzzlingly, these near-simultaneous deaths happen soon after the scientists witness the extinction of a rare species, the glowing winged mouse, in real time, standing next to each other, conducting a breeding experiment with its last two known members in secret. The prayers offered by the narrator are for the scientists, Dr Nobuhiko Akedera and Dr Keiichi Sakakibara, after the latter’s death. No longer able to speak or breathe without the help of machines, Dr Sakakibara has transmitted a deathbed testimony through ocular movements translated into a word processor. His efforts to communicate precipitate the pneumonia that finally kills him. A fictionalised Dr Ishiguro (the author also trained as a biologist) includes this report in the latter half of the story. Sakakibara’s report goes beyond the official version of the extinction released by the scientists to the media: in a shocking third act, he reveals that the female mouse was pregnant, that the fetus was alive in its mother’s body after her death, and that his colleague, the celebrated Akedera who came to Hokkaido to investigate this rare species, killed the baby mouse intentionally.

The mysteries of the glowing winged mice are multiple. There is the mystery of their anatomy, their internal organs positioned completely differently from rats, mice and other mammals: a heart without chambers and no reproductive organs. Then there is the strange light observed when two of them are in proximity with each other; their deaths at particular times of year; their long, possibly infinite life spans; the function of the odd little structures on their back, colloquially called ‘wings’; that their cell lines resist the scientists’ attempts to culture them; the ‘death switch’ which, when activated, sets in motion an irreversible course towards death; and perhaps most mysterious of all, the possibility that this switch makes biological reproduction possible. It becomes clear to the scientists that for this species of mouse, reproduction and death are closely linked. This spells trouble for the species when efforts begin to make the last two mate in a captive breeding experiment. The scientists are primarily occupied with finding explanations for these mysteries in the mice’s DNA; that is, at the level of the molecular.

In the English translation, the taut prose of the report is typed out in Courier New, a typeface first created in the decade of the double helix’s discovery (1953) and the publication of the Central Dogma (1957), dating the typography to the technological beginnings of molecular biology. In this graphic evocation of techno-scientific efficiency, Ishiguro gradually opens out alternative readings of reproduction from molecular explanations, to uncanny effect. As the report-story unfolds, it becomes more difficult to separate questions of methodological repetition from those of historical memory. That is, the viability and efficacy of certain normed procedures of scientific investigation from the damage the breeding experiment causes. While the events of the story seem to focus on biological reproduction—mating the mice and culturing their cells—these other forms of reproduction take on interpretative significance, suggesting alternative readings of data and circumstance that begin to encroach on the scientific oversights. As well as including fragments of remembered conversations, arguments and untested hypotheses, the submerged past of the island appears in grainy reproductions of local photographs, and incidental flashes of names and myths of Hokkaido’s Indigenous Ainu communities and their expelled cosmology. Not incidentally, given the author’s choice to give the author of the report his own name and qualifications, Tatsuaki Ishiguro was born in Hokkaido. This paratextual detail adds more hermeneutic anxiety when evaluating the story’s inconsistent references to Indigenous cosmology, imperial history and scientific modernisation: where does Dr Ishiguro stand? How does his own relationship with Hokkaido bear on the way he compiles the report, the details he decides to include, and the hypotheses he chooses to state or leave out?

The unevenness of these reproductions creates a sense of a scientific account placed under strain by unassimilated forms. At points, it seems that all these forms of reproduction might be significant in some way, but as the scientists race against the time of species death, the space for interpretation becomes increasingly constrained. This strain is conditioned by several intersecting tensions: between the historical and the biological, the local and the global, and most dramatically, the difficulty of reconciling the mice’s reproductive behaviour with a selectionist version of species survival. That is, between measuring the mice against model test subjects capable of reproducing, and encountering them as fellow organisms who seem to be programmed towards death. This latter realisation draws the living bodies involved in the experiment into closer proximity, levelling out the differences between scientific understanding and patterns of living matter, and rehearsing molecular biology’s ambivalent relationship with some of the more open-ended questions of evolutionary biology. At the edges of Ishiguro’s story is a strained encounter between lifeforms, formed in part by Darwin’s reception in 20th-century Japanese biology, by half-assimilated Ainu cosmology, and by the physiological proximity between the mice and the scientists in the latter’s attempt to preserve the species.

Through its representation of this strained encounter between historical circumstance, scientific epistemology and human and non-human lifeforms, Ishiguro’s story closes the distance between Hokkaido’s endangered species, its Indigenous history, Japan’s political economies of biological and cultural preservation, and the laboratory procedures that link these together. The fictional Species Preservation Centre is an ‘enhanced environment’ connected to a global biopolitical economy of hope around species preservation, whose methods are reproduced in the scientists’ captive breeding experiment. Drawing critical work on conservation and colonialism, and extinction studies and multispecies ethnography into a genealogy of laboratory studies, this article reads Ishiguro’s story alongside Hannah Landecker’s recent theorisation of ‘anthropogenic biology’ (2024). Landecker uses this term to describe changes in organisms produced by forms of human biological control, changes neither ‘anticipated by (this control) nor legible within its originating logics’ (2024, 2). For Landecker, this offers a way to move beyond the elegiac romanticism that often accompanies theorising the Anthropocene, and to narrow down the capacious biopolitical analyses of modernity’s effect on life processes tout court into

the novel patterning of living matter and processes across space and time, from the molecular to the ecological, that arises with forms of biological control and biotechnology and because of them. (2024, 4)

Landecker’s extension of biopolitics makes an epistemological case for the importance of analysing complex forms of metabolic adaptation. She does so by arguing that environments of biopolitical experimentation structured around outdated theories of biological causality have already transformed life processes. Whether or not the science is wrong and/or incomplete, its errors and/or incompleteness do not mean that it has not already had an effect on living processes and the biological make-up of new generations.

How is biovalue to be calculated in conditions of planetary emergency, beyond the actuarial controls of a political economy motivated by the reproduction of forms considered capable of reproducing? The scientists are confused and exhausted because they cannot work out the mice’s evolutionary value if they are, as it seems, programmed towards death. Around them, the surroundings of the story made up of other reproduced forms contaminate the enhanced environment of the laboratory, troubling the judgements of weakness and strength on which this preservation initiative depends. Landecker’s emphasis on patterning and proximity over selection and survival offers a critical accompaniment to Ishiguro’s rewriting of a familiar sf plot, from Mary Shelley’s *The Last Man* (1826) to Michael Crichton’s *Next* (2003): technologically enhanced reproduction at the point of species-death in (usually misguided) techno-scientific attempts to prevent or reverse extinction. For Lee Edelman, this plot represents a social reckoning with the death-drive which places the Child as ‘the perpetual horizon of every acknowledged politics, the phantasmatic beneficiary of every political intervention’ (2004, 3). This horizon repeatedly frustrated, the scientists encounter the mice as feeble creatures incapable of development, and the experiment becomes a decision over whether the species is valuable enough to preserve.

Srinivasan offers a way to interpret this decision as part of a general phenomenon in wildlife conservation, whereby human-centred values of well-being are applied to non-human populations (Srinivasan 2017). The language of decline and endangerment in conservation biology carries the charge of a world in which human survival is under increasing pressure. Fears around uncontrolled reproduction are often attached to necropolitical solutions: to curb these trends through enforced measures of population control, either through the slowly-violent preparation of societies for lower numbers of people, or faster, surer measures of population destruction (Nixon 2013; Biermann and Mansfield 2014; Mbembe 2019)? To adapt Carlos Novas’s (2006) concept of a ‘political economy of hope’ in this planetary context, these justifications constrain possibilities of reproductive justice for communities who fall outside core sites of mitigation and obscure the realities of environmental pressures caused by industrialisation.

In Ishiguro’s story, these planetary politics are plotted through the short-term fellowship between the two molecular biologists and their encounter with these anomalous lifeforms in the laboratory. Landecker’s argument about the effects of fallible causes has a catastrophic analogue in the story: the extinction of the mice is brought about by the scientists’ mistaken assumptions about the mice’s life processes; specifically, in the mystery around how the mice reproduce. Their final secret experiment is conducted under the light of a single lamp, in a hyper-focused, closed environment. This is not because they think this will improve the mice’s chances of reproductive success, but because they do not want to be discovered, and they also do not want to miss out on observing any unusual phenomena. The anomalous, though not unprecedented result of the experiment—a gestation at the point of death—as well as their shared failure to communicate this result shines a spotlight on both scientists’ inability to anticipate the effect that their own control over the environment has had.

The report story is full of strain, of method, physiology and emotion: the forced reproduction and its effect on the mice; the sieving of bodily substance into molecular data; the pressure to communicate; the physical demands of the experiment and the tightness of its technical and epistemological constraints; and the final movements of the baby mouse as it attempts to begin life, ‘trying to get to its feet’ (99). This article tracks the strained encounter between biological reproduction and extinction mitigation in Ishiguro’s story, in which the controlled attempt to manage the mice’s extinction in the enhanced environment of the laboratory results in destroying the species, and, it seems, the biologists. The hubris of this ending is relevant for futures of human reproduction in the context of global environmental damage because the ethical investments in biological conservation in and out of scientific contexts often allude at once to the disappearance of human populations as a result of environmental crisis, and to anxieties around global overpopulation and its ecological harm. I suggest this strained encounter as a genre of anthropogenic biology; that is, as an imaginative condition of life as aftermath. Ishiguro’s story demystifies the extinction it dramatises. It moves the mystery of the glowing winged mice’s extinction from science fiction to scientific realism, subverting the plot of species-death through the immediate encounters between different forms of living matter documented in the report. What emerges is not so much a narrative of extinction anxiety, but a story about how the disturbance of species identifications can open the most closed scientific spaces to novel patterns of communication between lifeforms. The tragedy of a cancelled future plays second fiddle to ‘the second crop and that which feeds on it’ (Landecker 2024, 5): patterns of life continue to emerge in these encounters.

## Endangerment and indigeneity

DNA has become an uncanny object of modern science in that its propagation relies on the heimlich a priori of cell division—the process of organic reproduction—while it is also figured as a timeless substance that migrates between the bodies it programmes. Japan’s participation in the Human Genome Project, one of the six nations that contributed research infrastructure and data to it, followed a century of Darwinism being used as an instrument of Japanese modernisation. Ishiguro’s story was published when DNA sequencing was still considered a primary potential technology for modernising human futures through the extraction, replication and analysis of biomatter, and alongside this, for capturing the past in molecular form.

Ishiguro draws in questions of reproductive futurism by turning back to the submerged history of Hokkaido’s declining Indigenous communities, the island’s main inhabitants until the late 19th century. Until 1869, the island was named ‘Ezochi’ in Japanese (‘land of the barbarians’). The island’s occupation, annexation and colonisation by Japan’s Meiji government was followed by a forced assimilation programme for the Ainu who lived on the island. This was carried out through land dispossession, the prohibition of Ainu practices and ‘breeding out’ Ainu communities. Ainu men were forbidden to hunt and fish, Anglican missionaries established conversion programmes on Hokkaido, and Ainu matrilineal family networks were destroyed. The history of Meiji policy towards the Ainu falls within existing definitions of settler colonialism as defined by Fayez Sayegh and Patrick Wolfe: discriminatory racial policies, violent actions and expansionism (Sayegh 2012, 21); and a system perpetuating the erasure and destruction of native people, and the expropriation of lands and resources (Wolfe 2006, 387). The Ainu became subjects of cultural conservation in anthropological studies from the late 1970s, but scientific interest in Ainu genetics came later, with investigations on maternal and paternal Ainu lineages to determine their genetic origin carried out in the early 2000s (Tajima *et al*. 2004). This scientific interest in their genetics was preceded by a surge of national interest in their practices and world view in the 1990s and governmental support for Ainu cultural preservation, much of which post-dates Ishiguro’s story.^1^

The language used by Dr Ishiguro to describe Hokkaido echoes that of naturalists and surveyors. He describes the area of Hokkaido where the Species Preservation Centre is constructed as a ‘nearly unexplored area surrounded by virgin forests’ (14), a *terra nullius* locality. Kamuikotan, which is also where the mice are found, is an Ainu word which translates to ‘gathering place of the gods’, the place where humans go after death (14). Ainu influences appear in single phrases or tales that have been passed down, but there are no Ainu characters in the story. These tacit references to the Ainu, while marginal, position the laboratory in a centuries-long history of colonial encroachment on the island and its modernisation over the 19th and 20th centuries. The narrative proximity that Ishiguro creates between the mice’s extinction and Hokkaido’s disappeared Ainu communities pulls both into the timeline of post-Meiji modernisation.

Ishiguro registers this encroachment through the minutiae of decision-making in the Species Preservation Centre, slowing the events of the story down and pulling actions out from compressed summary, up to the moment when Dr Akedera holds a pair of heavy scissors over the body of the baby mouse and drops them on the creature, killing it, while Dr Sakakibara looks on in disbelief:

*By the next moment the baby mouse already lay crushed in the transparent amniotic fluid.*

*As translucent organs poked through its torn skin I wondered what had just happened.*

*At first I thought he had dropped the scissors by accident, but Akedera-san’s expression remained unchanged.*

*I was speechless.*

*I could not believe my eyes.*

*No matter what I said, Akedera-san would not answer me.* (100)

The fragility of form and the immediacy of description reconstruct the moment of species death through a grotesque sequence of image and action. The italics recreating the translation of ocular movements into speech stand in for the physical effort of communicating this event, paragraph breaks indicating pauses between eye movements. The shuddering arrangement of this sequence brings the dying scientist’s body struggling to communicate into proximity with the crushed body of the mouse lying on a lab bench, implying a simultaneity, even a contagion, between their deaths. Dividing them is the ‘unchanged’ expression of the familiarly-titled but estranged ‘Akedera-san’, who gives no explanation (the ‘san’ suffix means dear or honoured, as if Sakakibara crosses this remembered gulf to hold Akedera close in the embrace of a superfluous word by the eye-muscle). Dr Akedera’s unchanged expression is an axis of structural condition, ‘remained’ suggesting a moment of realisation, the coming-to-pass of a fixed outcome, not a narrative shock. Earlier in the story, Dr Sakakibara admits in his interview that it seemed that “Dr Akedera had some idea of how the story was going to end” (84). This sequence demonstrates that this prescience was more over an inevitable course of action than what Evelyn Fox Keller calls a ‘feeling for the organism’ (1983).

Nonetheless, there is a great deal of feeling involved. In the build-up to the baby mouse’s death, the report records the sensorial responses of the scientists to the mice and their information, the psychic and physical strain it causes each of them, and how Dr Sakakibara’s more cautious approach puts pressure on his relationship with Dr Akedera: ‘The relationship between Drs Sakakibara and Akedera would become rather strained after this’ (65). The disagreements between them are frequent, and Akedera does not give way to his colleague easily. Dr Ishiguro begins to suspect that the latter’s motivation for carrying out the research was ‘a fear of death’ (65). In his report on failing to culture the cells of the mice, well before his killing of the baby mouse, Dr Akedera questions “‘these feelings of superiority and inferiority that the living choose to habour regarding the dead … Words like “extinction” and “death” betray the self-centred logic of the living”’ (65). The failure to cultivate allows him to speculate on misunderstandings and incomprehension. The strain of his encounter with the mice prompts a one-sided fellowship motivated by the apprehension of his own fear of death.

One of the more curious aspects of the story is its representation of fellowship: being someone’s colleague, or cage partner, or sexual mate, or companion in death; someone or something with whom one shares interests. These social relations are not as easily managed as a laboratory’s environmental conditions. In *Laboratory Life*, Bruno Latour and Steven Woolgar discuss a sentence written by astronomer Fred Hoyle in an otherwise empirical description of the discovery of pulsars, a type of neutron star. While the discovery was first reported in February 1968, it had been discovered by a PhD student, Jocelyn Bell, during ‘a 2-month period up to September 1967’ (Hoyle, quoted in Latour and Woolgar 1986, 35, 1979). Hoyle’s note of a delay between discovery and report makes no difference to Bell’s proof of pulsars’ existence, but it does suggest something to Latour and Woolgar about the unknowability of what may or may not have been revealed by Hoyle’s observation about the process of discovery. They wonder if this ‘utterance’ might suggest ‘the existence of a complaint’ that the research group had ‘somehow violated scientific protocol by delaying news of their discovery’, hindering the progress of other teams (35). Alternatively, it could be an utterance of admiration, ‘again because the time period is a noteworthy or unusual feature’, and because the discovery was made by a PhD student whose team needed time to work ‘unhindered by outside interference’ (35). The large number of alternative readings of Hoyle’s utterance is part of Latour and Woolgar’s broader argument that the social phenomena of laboratories are entwined with the processes through which scientific order is constructed out of chaos. The representation of scientific fellowship on the pages of reports matters for the way scientific fact is established, and how it might be contested.

In ‘Deepest Sincerity’, the glowing winged mice become extinct, but not in the way the two scientists report the extinction. The initial omission of the baby mouse’s existence comes about in part because of the increasingly strained relationship between the two biologists as they race to save the species. In Dr Sakakibara’s account, Akedera’s insistence on secrecy ends up throwing the experiment into chaos. Their official versions of the extinction are mined by the local newspaper for quotes that invoke an elegiac romanticism for lost species to which Landecker offers the alternative of anthropogenic biology. Dr Sakakibara: ‘“I sensed the moment when a species’ energy, spanning millennia, was no more”’ (94-85). Dr Akedera: ‘“I found myself thinking upon the many species of flora and fauna that vanish every day from this world”’ (95). There is a striking asymmetry in Dr Sakakibara’s statement between the numerical phantasm of an energy that spans the deep time of ‘millennia’, and is then witnessed by humans becoming ‘no more’. As Luke Donahue argues, this ‘possibility of witnessing the moment of extinction is entirely unprecedented’: witnessing here invokes the real-time annihilations that are now possible to track by piecing together an enormous volume of information about the histories and decline of species (2021, 945). Dr Akedera’s ‘thinking upon’ flora and fauna vanishing is conspicuously vague in stating what or who might have caused this disappearance, but ‘from this world’ could allude to a circumscribed sense of place: a world for which humans might take, or have taken, responsibility. These statements indicate a performance of imprecision, of mulling on a problem, or of the fallibility of scientific endeavours, the scientists coming out of the laboratory to mourn the loss, and their failure, in public. When the truth of the extinction is revealed, Dr Ishiguro’s analysis pulls the story back into a tone of expediency.

In a comment that echoes Latour and Woolgar’s inconclusive speculation on a delay between discovery and report, Dr Ishiguro notes,

In the final analysis, nothing that can be said about the incident escapes the realm of speculation. We are left with no option but to think that Dr Sakakibara’s comment—‘If one mouse did remain, what could ever have come of it?’—says it all. (101-102)

Dr Ishiguro the narrator is less careful than Ishiguro the writer, or than Latour and Woolgar the sociologists, to leave space open for alternative readings. Dr Akedera’s decision was a pragmatic one. With no known mate, the mouse was the last remaining, and so the species was already extinct at the point of its conception. The experiment failed at the point the scientists failed to keep the baby mouse’s parents alive, and in the failure of its parents to produce a litter. Dr Ishiguro’s speculation anticipates a question that has become central to extinction studies: when does a species become extinct (Adams 2004)? In the strict typologies of classical evolutionary biology, it would be when it cannot reproduce itself, but in complex accounts of evolutionary survival put forward in multispecies ethnography which reject the idea of species as distinct, separate types, an alternative version of biological interdependence would allow for a version of species difference as complex entanglement (Kirksey and Helmreich 2010). This position shares ground with a critique of postcolonial studies by settler colonial studies: by positing extinction as a ‘culture object’ which reinforces a hierarchical catalogue of species, the extinction concept ends up reifying the system of knowledge environmental activists should resist, as Ursula Heise has argued (2016). Donahue considers these positions together to suggest a species-based concept of extinction that also acknowledges what Deborah Bird Rose describes as ‘the death of temporal, fleshy, metabolic relationships across generations and species’ (Rose 2020, n.p.). This would keep the ecocritique turned towards biological exhaustion and necropolitical infrastructures and avoid the susceptibility of extinction-as-survival to the spurious denialism of nature fighting back (Donahue 2021, 941). Donahue’s critique parallels Landecker’s insistence on an anthropogenic biology. It distinguishes between epistemic justice (countering a selectionist species-thinking) and political justice (countering the historical occlusions of industrial ecocide), and considers how these different registers of environmental justice might work together.

Ever the model colleague, Dr Ishiguro offers an alternative reading of this debate. Within the narrowing space between scientific realism and science fiction, his interpretative gesture only invites further confusion. While Dr Sakakibara’s interview solves some of the mysteries of the winged mice, he writes, ‘others have remained unresolved’ (102). Dr Ishiguro cannot help involving himself in the investigation, taking the report on a ‘detour’ towards something that caught his attention in the final scene, a note in Akedera’s journal, but which Sakakibara does not mention: the electrocardiography data of the two mice ‘in the last moments’ (103). Instead of the sharp peaks of vertebrate species, the mice’s heartbeats form ‘a gentle sine curve’ (104). To understand this, Dr Ishiguro reintroduces a character who appears briefly at the beginning of the story: the retired biologist Dr Ishikawa who stumbled on a winged mouse while on a hike in Kamuikotan, performed the first recorded dissection of the mice, was the first to publish on their anomalous anatomy, and who suddenly ‘went missing (while mountain hiking, it is said, though the details are obscure)’ (19). Dr Ishikawa had suggested that the mice have ‘a heart structure that lacks chambers’; to take this literally, this means that the usual operations for managing the flow of oxygenated and deoxygenated blood around the bodies of mammals (and birds, and crocodiles) through four chambers happen in one big chamber. The time of the glowing winged mice’s bodies, without the ‘pulsing waves’ of mammalian heartbeats, is slower, longer, and in this ‘hibernation-like state’, possibly infinite (106). This is not a noisy, violent version of evolution where organisms respond to selective pressures, or a more anarchic version of horizontal variation across ecosystems. It is akin to an evolution of continuity using the bare minimum of energy, more a bodhisattva practice of sitting still than a New Testament evangelical graft of spreading the will and word of God, or a disposition towards accumulation and growth.

The glowing winged mice seem to possess a biological function that prevents fast population growth. At the moment of sexual reproduction, the scientists observe, some kind of ‘death switch’ must be activated that causes the mice’s bodies to degenerate quickly and irreversibly. When the species becomes extinct, this death switch has been activated in the laboratory as a direct result of the breeding experiment. The death switch has a historical precedent in the imaginative history of evolutionary biology, appearing in colonial biological writings through a genealogy of deathly landscapes, where evolutionary ‘ancestors’ incapable of development emerge from dark and uninhabitable landscapes.

## The child, the native and the genetic ark

Biodiversity laboratories have an odd double life. They are sites of enclosure which bring in resources—biomatter, scientists and equipment—to increase both the value of their scientific credibility and their capacity to attract further investment and funding. They reproduce the technological apparatus of global molecular biology, enabling the transnational exchange of data and information while expanding the reach of its influence in new, unexplored and scientifically underdeveloped areas. As Karin Knorr Cetina puts it, laboratories ‘provide an ‘enhanced’ environment that ‘improves on’ natural orders in relation to social orders’ (1999, 26). When it comes into the laboratory, the life process is converted into an experimental object, separated from its external context and reduced to ‘partial versions’ to be ‘manipulated … on their own terms’ in often-accelerated reproductive cycles (Cetina 1999, 27). Laboratories like these are designed to operate within a narrow, global vocabulary, distinct from and incommensurable with the lifeworlds and systems of knowledge just outside their walls.

The Hokkaido Species Preservation Centre is an enhanced environment whose ambition to breed the rare species in its care, under threat of extinction in the wild, leads to controlling their environment in the laboratory: temperature, lighting, food, sleep patterns, as well as providing them with sexual mates. Despite this control, the Centre is caught in a more local web of influences than the global scene of molecular biology it reconstructs. As shown by Dr Ishiguro’s inclusion of Dr Ishikawa’s heart-without-chambers theory, these local attachments mean that forms and practices of interpretation that fall outside the molecular become part of the process of scientific deduction in the story. The Centre is constructed on the site of an old elementary school. In photographs, Dr Akedera notices too late, the mice appear near children, and Dr Sakakibara’s last words were ‘“the child’s” with no context’ (102). When two mice are in proximity, their ‘wings’ emit a faint light—a ‘glow’—and on observing this for themselves, the scientists decide that this must be a form of communication between the mice. When the data on the patterns of light are sent for analysis to the Tokyo University Centre for Languages, an assistant describes them as ‘wave form peaks that resemble a musical scale when digitised’, but concludes that they do not meet the criteria for a language. Assistant Kobayashi describes the glow in these terms:

‘Some typical patterns reminiscent of infantile utterances were identified, but a homological comparison with existing languages reveals their inconsequential poverty.’ (85)

These words arrive from the techno-scientific core (Tokyo) to the experimental periphery (Kamuikotan) already estranged from their context. Kobayashi only has the wing light data to go on; she has never seen the mice, and she is a linguist, not a biologist. Nonetheless, her analysis takes up a page of Dr Ishiguro’s report, and the mice’s communication style is compared with and dismissed from a corpus of global languages. The repeated references to children mark a symbolic split in the story: they anticipate the pathos of the baby mouse’s death and invite condemnation of Dr Akedera’s final act. It places the mice in an imaginary world of infancy and playfulness, carrying the energy of newly-arrived beings. These references to children also invoke a moral imperative to save the species, because as Edelman puts it, the Child is the subject for whom ‘the telos of the social order … is kept in perpetual trust’ (2004, 11). This immanent symbolism is undercut by historical intertexts of infantilised natives and keeps the history of Hokkaido’s colonisation at the surface of scientific interpretation.

The glowing winged mice of Ishiguro’s story become subjects of species conservation when their numbers begin to fall. This fictional decline coincides with the real decrease in Hokkaido’s Ainu population numbers in the late 20th century, while anthropological interest in them was on the rise. Assistant Kobayashi’s judgement about the form of communication represented by the wings’ glow is converted into empirical fact through a pre-existing hierarchy of languages. The Child and the Native sit at opposite ends of a developmental chronology and evolutionary hierarchy that ensures investment in selectionist conservatism: futures which must be preserved, and pasts that have already been cancelled. The mice go from one end of this telos to the other: from the Child who must be preserved, to the Native incapable of ensuring their own survival.

A Conrad Martens watercolour painting from 1833 shows the HMS *Beagle* sailing into a shallow lagoon, greeted by people in canoes either waving or wielding spears, with no Europeans in sight on deck or shore. While Martens’s painting imagines the scene of European arrival from the shore of Tierra del Fuego, his ship-mate—a young Charles Darwin—introduces this location by recalling his foray into the dense forests on the banks through the ‘entangled mass’ of ‘irregular masses of rock’, surrounded by decaying and torn-up trees. He writes of this encounter: ‘Death, instead of Life, seemed the predominant spirit’ (2009, 222). What follows is a much-analysed sequence of descriptions in *The Voyage of the Beagle* on the physical appearance and customs of a composite Indigenous group that the colonialists call the Fuegians. Gregory Radick argues that Darwin’s writing on the Fuegians sets the tone for his later writing on human evolutionary lineages in *The Descent of Man* (Radick 2010), while Noah Heringman describes it as,

something of a palimpsest, incorporating both the wonder and philosophical reflections of the earlier accounts [of the Fuegians] to produce an intertext that remains an active inheritance for the late Victorian science of human origins. (2023, 185)

Heringman interpolates this scene with the eternal recurrence of an encounter between European colonial endeavours of discovery, cartography, trade and profit, and the people and landscape of unknown lands from Europe’s ancestral past. There is an incommensurability of motive in this encounter: the baffling customs of the Fuegians, their survival in these hostile surroundings, and what seems to Darwin to be a more proximate relationship to death. ‘Viewing such men, one can hardly make oneself believe that they are fellow-creatures, and inhabitants of the same world’, he writes (Darwin 2009, 225). To Darwin, the Fuegians traverse a border between the world from which he has arrived, the world of Life, and Death, the world of the thick, infernal forest in which he does not allow himself to remain.

Darwin transmits the charge of the encounter by writing it down. He dramatises the limits of his interpretative capacities; he cannot make evolutionary meaning out of the Fuegians beyond their ancestral resemblance to Europeans and reduces their endeavours of survival to an incapacity to develop. The ship becomes a space of retreat to which Darwin and Martens return to document their respective impressions and observations, and Darwin converts his physiological responses (of fear, of horror, of confusion) into a voice of judgement enclosed by the floating vessel which will soon be on the move again.

In a discussion of biodiversity models of environmental justice restricted to captive breeding, Malcolm Ferdinand reverses the relation between the entangled forest-as-death and ship-as-life and imagines a different subject observing the arrival of a foreign ship: ‘From the shore, it is not possible to know with certainty whether this ship is a slave ship or not’ (2019, 192). The real catastrophe, he argues, is on board. Through a comparison of ecological crisis management with a Noah’s ark model of survival, Ferdinand offers a generative segue between a biodiversity model of captive breeding and the long history of biovalue amassed through the slave trade. An ark model ‘testifies to the refusal of the world … an absence of a world’, where ‘the dehumanisation of captives, chaining them up in the darkness of the hold and the steerage, means that a world cannot be established on this ship’ (194). In the refusal of the world, there is no substitute for life processes, only the narrowing down of life into closed environments.

Ishiguro regularly takes the mystery of the mice out of the laboratory and charges it with symbols of frontier terror. Dr Ishiguro notes that winged mice were spotted by a policeman on a nearby vista called Indian Peak, halfway up Kamuikotan’s mountains. The name was ‘inspired by Westerns, where North American Indians fall on stagecoaches on cue from a lookout’ (47). A globalised gothic accompanies the imported scientific infrastructure, in which Indigeneity figures as a peripheral threat of retribution from vengeful spirit-men. This, the story suggests, is the phantasmal threat against which biodiversity initiatives defend their specimens, and responding to this threat means containing life on board, in the laboratory. In managing the tension between life as process and life as genetic ark, biodiversity preserves a typological ideology of discrete species and subspecies, which might say more about colonial-capitalist abbreviations of life than the way life processes transform over millennia.

## A science of lost data

To make futures of human reproduction just and equitable in the face of political and planetary challenges to social and ecological cohabitation, Ferdinand calls for ‘a concern for living together’, set against the ‘no life on board’ of ark-based survivalism (2019, 194). Nonetheless, species-thinking—and with it, sexual reproduction—still dominates legal and scientific models of environmental justice and calculates biovalue in terms of reproductive capacity. When species begin to lose numbers, those forms that cannot reproduce enough on their own can be preserved as genetic data. In 2000, a group of molecular biologists published a paper in *Science* promoting the preservation of endangered species’ DNA. They frame this as a loss of data: ‘the loss of biodiversity resulting from extinctions’ is accompanied by a ‘decrease in access to genetic resources’ (Ryder *et al*. 2000, 275). The conservation solution should be the institution of biobanks around the world to store genetic data in a kind of molecular ark. Among this group was Sydney Brenner, one of the central figures in the development of molecular biology. In the early 1960s, Brenner worked on an experiment with Francis Crick, Leslie Barnett and Richard Tobin that would show that the genetic code comprises codons that programme amino acids: three base pairs for one amino acid, the building blocks for protein (Crick *et al*. 1961). Their model organism was a bacteriophage, a bacteria-killing virus that looks like a long-necked spider, and a standard model organism in genetic research. Standard partly because it is so versatile and partly because it is very easy to find; a prolific, widely available biological ‘entity’, easy to preserve and easy to replicate. They found that the genetic code has a triplet organisation: three base-pairs for each acid. This is not just true for bacteriophages; it is (nearly) universally applicable (Koonin and Novozhilov 2009). This powerful description established the basis for a range of scientific procedures grounded in the assumption that this organisation of code was (and is) universal. That is, it establishes the possibility for universal measures of calculation in molecular biology. It is also an example of how lifeforms with fast reproductive cycles become so useful in molecular biology, and subjects of preservation as model organisms in big science (Ankeny and Leonelli 2011, 2013).

With their slow, confounding reproductive patterns and the resistance of their cells to cultivation, the glowing winged mice are the antithesis of bacteriophages. The seeming absence of virile behaviour, fertile potential and replicable matter eclipses research interest in their anomalous constitution. The accumulation of evidence that proves their weakness transforms them into objects of threat, not potential sites of profit. Recent work on the relationship between ecological conservation and colonialism has shown that conservation initiatives often involve displacing or eradicating ‘troublesome’ existing populations, human and non-human (Crowe and Shryer 1995; Adams and Hutton 2007; Probyn-Rapsey and Lennox 2022). Jevgeniy Bluwstein has called this ‘the double movement of colonising landscapes/landscaping colonies’ (2021, 904). These practices point to correlated infrastructures of funding aims, scientific method and the dispossession of a continuum of human and non-human inhabitants of profitable land, where the teleology of Child and Native can be adjusted according to speculative calculations of risk and loss. Read through a history of deadly conservation practices, the spectacular quality of the baby mouse’s death is not the result of a sleepless, harried scientist losing perspective, but, as Dr Ishiguro suspects, the pragmatic realisation of an extinction that has already happened within a tightly selectionist rationale. It reverses the teleology of reproductive futurism, so that the Child of the reproductive future (the baby mouse) becomes the Native incapable of development (the Indigenous Ainu).

The values placed on the futures of human reproduction as a species are intimately tied to the devaluation of non-human life as life extrinsic to species survival. Conservation biology operates through strained encounters where decisions are made to determine what and who should have a future, and what and who constitute a threat to this future. This model of conservation depends on privileging what Srinivasan identifies as a collectivist rationale of species, populations and ecosystems over individuals, or individual encounters between lifeforms. In place of this, she proposes that wildlife conservation goes back to its roots: ‘the flourishing of non-human life’ (2017, 1459). She notes a utilitarian scale between the conservation and reproduction of human and non-human life which indicates ‘the embedding of human-centred values and assumptions relating to economic development which are otherwise incompatible with the goal of non-human well-being’ (1471). This interpretation of conservationism has implications for the futures of human reproduction because any attempt to think against species models of conservation means paying attention to survival practices that do not privilege sexual reproduction as a grounding principle of species classification. Cladistic and genetic species classifications are based on the principle of common ancestors, and therefore, on relationships of sexual reproduction. These classifications are brought to bear on the determination of a particular species’ evolutionary success across long spans of time. To put it another way: ark-based survivalism is an addition to, but not a replacement for, values of human survival.

If, for Landecker and Ferdinand, hope for environmental justice beyond scientifically justified ecological destruction resides in opening the closed world of the laboratory out to the worlds outside it, then Ishiguro’s story hints at an oblique and provisional reparative mode in the fusion of spirituality and Darwinian science in post-Meiji Japan. The space Ishiguro allows for alternative readings of the glowing winged mice’s extinction not only demystifies the extinction, but contextualises the event through Darwinism’s varied reception in Japan over the 20th century. This variation in Darwin’s relevance for and to evolutionary scientists and philosophers in Japan offers a counterpoint to the direct import of molecular technologies and ideology represented by Dr Akedera’s status as a world-famous biologist from Tokyo. The story implicates encounters both fraught and generative between scientific, theological and cosmological versions of life processes; specifically, between Darwinian evolution, Mahayana Buddhist materialist philosophy and Ainu cosmology.

## Strained encounters of the anthropogene

The mystery of the glowing winged mice’s extinction is solved in the story’s third section, when Dr Sakakibara uses what energy he has to tell Dr Ishiguro about the final, secret experiment conducted by the two scientists: Akedera’s proposal to attempt once again to breed the mice. Sakakibara seconds this proposal, which he ‘recognised as criminal, and regretted until the moment of his death’ (76–77). Akedera wants to determine whether the simultaneous deaths of the other winged mice were the result of an infectious disease. The scientists place the two mice, given the Ainu names Ai and Ponta, next to each other in separate cages and a plastic divider between them, to ensure that they can ‘become aware of each other’ without bacteria and viruses passing between them (77). At the level of description, the two mice are bracketed from each other by cage, plastic and the hopes of the scientists, while their own awareness of what is happening—and desire for what might—is illegible in the report.

At first, nothing happens, and the mice appear to be indifferent to each other. Then, after a few days, their wings start to emit a faint light—something that has never happened when they are alone—and this ‘glow’ does not show up in the photographs Akedera takes using high ISO and infrared film; that is, its image cannot be reproduced via high-speed or electromagnetic photography. Then, a clear liquid starts leaking from their eyes which on analysis is determined to have the same composition as normal mammalian blood. Dr Ishiguro’s report shows how the scientists measured the degree of strain:

But for a winged mouse, which weighed barely two hundred grams, a day’s worth of tears equalled about a quarter of its bodily fluid mass, and ten sub-dermal injections meant replacing all of its blood.

‘Looking at those tears, I wanted to cry myself,’ Dr. Sakakibara recalled about that period. (83)

Here, the complex problem of equivalence between living beings is negotiated by a teleology of weakness to strength. ‘Barely’ is followed by ‘equalled’, and then by ‘replacing all’: the mouse’s total weight is converted to a calculation of fluid mass, supplemented by synthetic liquids. The emphasis is not on what is, but on what can be done. On its own, the mouse is a slight creature under strain from the volume of its fluxes. Dr Sakakibara’s comment restates the problem of equivalence in an ambivalent declaration of affinity—“I wanted to cry myself”—hovering between rhetorical sympathy and being on the verge of tears. If the former, it indicates the scientist’s frustrated labours; if the latter, a sensorial response that suggests his proximity, or his desire to be close, to the mice.

As Ai and Ponta leak out life, Dr Sakakibara suggests putting them in the same cage—‘an undivided chamber’, writes Dr Ishiguro. This description displaces the anatomy of the mice’s chamberless heart structure onto the experiment’s final section, as if their bodies have begun to shape the procedures, rather than the other way round. Their feelings are now forefront in Dr Sakakibara’s hypotheses: he speculates that the tears are ‘a lamentation that they could not be together’ (84). This move makes no difference, because the mice continue to shed tears (Dr Ishiguro avoids using words like ‘cry’ and ‘weep’ in his report). Their loss of fluid is clearly killing them, and the intravenous injections damage their internal organs. There is no possibility of life support. Their proximity—being ‘face to face’ with each other—seems to have activated an irreversible ‘death switch’ (88). The scientists are exhausted and desperate to work out what is happening, and why. Is there a link between procreation and death, in this species? This is one of Akedera’s theories, comparing it to the way the HIV virus survives, while Dr Ishiguro speculates from the safe distance of the report, ‘It would not have been strange if Dr Akedera had begun to view his own narrative as the reality’ (90). A selfish gene would make a death switch that preserves genetic matter while killing its host the only possible reality. Finally, the two mice die within hours of each other.

Earlier, this article compared the sine wave structure of the mice’s electro-cardiac activity, and the hibernation-like state in which Dr Ishiguro speculates they spend most of their existence, to a bodhisattva practice of sitting still. This fusion of Buddhist meditation with Darwinian survival has a precedent in Japanese evolutionary biology. Darwinism arrived in Japan through a series of lectures given at the University of Tokyo by an American zoologist called Edward Morse in 1877, nearly 20 years after the first edition of *On the Origin of Species* (1859) and less than ten years after the Meiji colonisation of Hokkaido. Histories of science in Japan have tended to view this as a direct import of Darwinism, and particularly Darwinian selection, as a justification for modernisation. Among individual scientists, philosophers and theologians, however, the reality of Darwin’s reception was quite different, as G. Clinton Godart has argued. Godart offers an alternative history that saw Japanese biology recalibrate the relationship between the scientific and the spiritual, nature and God, and humans and non-humans over more than a century (Godart 2017). To keep Buddhism and Buddhist practices relevant in a society modernising at pace, with evolutionary theory part of its index of modernisation, there were several attempts to reconcile Buddhist materialist philosophy with Darwinian scientific materialism.

This produced epistemological compromises that joined Mahayana Buddhist ideas of the continuity and interrelations between humans and non-humans to Darwin’s idea of entanglement and adaptation in *On the Origin of Species*. In this compromise is the figure of a radical, ecologically minded Darwin, looking horizontally across the tangled bank, surrounded by plants looping together on the ground, birds in the bushes, insects ‘flitting about’ and ‘worms crawling through the damp earth’, reflecting on how

these elaborately constructed forms, so different from each other, and dependent upon each other in so complex a manner, have all been produced by laws acting around us. (2009, 429)

Here is proximity between organisms, and between organisms and their surroundings. While for Darwin these laws lead to the struggle for life and the extinction of ‘less improved forms’, the Japanese mycologist Minakata Kumagusu drew the entangled bank metaphor into his work on slime moulds and their ambiguous taxonomical classification. In a biography that bears more than a passing resemblance to the disappeared Dr Ishikawa, Minakata published in *Nature* but lived in the woods of Wakayama, south of Osaka. Godart describes him as ‘a maverick and freethinker, but also a careful scientist, a systematic collector of specimens and artefacts, as well as an ambitious religious thinker’ (2017, 93). Minakata thought that because of their physiognomy and eating habits, slime moulds should be classified not as fungi but as animals: they begin life as single-celled organisms which coordinate with each other, fuse and become a plasmodium, ‘one gigantic cell with multiple nuclei, or multiple cells without membranes in between’, a body capable of movement that leaves a mould behind it, and which then eats bacteria (Godart 2017, 95). Observing slime moulds in his garden over a 20-year period, Minakata concluded,

Without human help, organisms naturally change into new species, or are changing and unstable … One can see that in this wide universe, without the help of human intervention, there are constantly innumerable changes happening. (in Godart 2017, 96)

While Minakata’s observations of slime mould raise radical questions about biological morphology and ontology, these lines also draw out a historical context for a more complex account of speciation, based on a fusion of Buddhist transmigration and Darwinian entanglement. Read this way, Minakata’s is a methodological appeal against rational intervention in the evolutionary processes of non-human subjects.

Ainu cosmology does not consider plants and animals as independent species, but as deities. What does it mean to flourish in a cosmology where mortal and immortal entities cohabit, and where survival moves between organic and metaphysical forms of existence? The gods gathered in Kamuikotan are deities of non-human life-forms. Ainu cosmology has a classification system for the world: Ainu-moshir (the Human World), Kamui-moshir (the Divine World), the Pokna-moshir (the Lower World), Teine-pokna-moshir (the Dark and Damp Lower World) and Chikap-sak-moshir (the World Without Birds). Emiko Ohnuki-Tierney notes that moshir or mosiri (universe or world) means ‘the total sphere of Ainu physical and metal activities and phenomena,’ a land of living and dead Ainu, deities and demons (1973, 285). In Ainu-moshir, humans, animals and plants live alongside supernatural beings, benevolent and malevolent alike. Kamui-moshir is also a composite place: a world for heavenly deities, a world for Ainu-moshir deities, and the world after death for humans. The concept of kamui goes beyond animism, as Takako Yamada argues in *The World View of the Ainu*. Unlike animism, where spiritual existence is attributed to an entity (animate or inanimate), deities are part of everyday life in Ainu-moshir, created within and part of the world of the living, while distinct from living beings (Yamada 2001). Kamui stands in for a supernatural world.^2^ Ainu worlds are not infinite, and mosiri does not go beyond the territory of southern Sakhalin, the sea around it and the sky above. The Ainu universe is analogous to a human body, and the finite lifespan of a universe has two stages: the beginning and the present (Ohnuki‐Tierney 1980*,* 285).

These proximate theories of living processes in Hokkaido place more pressure on the encounter between scientist and mouse and exceed the report’s allegiance to selectionist materialism. This pressure arrives through utterances throughout the report that do not change its principal facts, but which open out multiple versions of circumstance and event. There might be a Minakata-like appeal to non-intervention against the injustice wrought on the mice, whose anatomy more closely resembles unclassifiable slime moulds than what might seem to be their more obvious kin: the common mouse and model organism, *Mus musculus*. Or could the report inhabit Ainu-moshir, where plants, animals and humans cohabit with deities, a world held together by measurements of time that span the life of a universe, not a species, in a circumscribed geographical locality? The report offers little in the way of consensus, only these residual strained encounters between theories of life process, between lifeforms, and between living processes and what is made to constitute their surroundings.

This article has considered futures of human reproduction in situations of planetary emergency through the analogue of captive breeding in enhanced environments. It has argued that anxieties around human overpopulation and its threat to non-human ecosystems have historical links with imperial fears of deathly, uncultivated landscapes inhabited by populations that seem to have little or no capacity for development. Biodiversity models of species preservation in life’s aftermath are often constrained to closed worlds of decision-making which privilege a model of life as genetic ark, rather than as complex process. Ishiguro’s story does not foreground sexual reproduction or reproductive strength as an index of life flourishing. Instead, the story alters the scientists’ relation to their surroundings, with questions of evolutionary significance, with their sense of their own bodies in a selective band of weakness and strength, and with their proximity to death switches and deathly landscapes. For Landecker, thinking of life as aftermath does not mean that life has come to an end:

For the ‘after’ is not an end time at all, it is more of life that goes on metabolising and dividing; not a problem of flourishing, rather a question of what flourished at which time, and what kind of problem that presents to contemporary society, politics, technoscience, and social theory. (2024, 5)

Landecker’s suggestion to consider flourishing not as a universal, nor as an aetiological mystery, but as historically bound to particular times and places—‘what flourished at which time’—describes a world of ‘hubris gone mouldy’: an anthropogenic biosphere (Landecker 2024, 1). This version of life as aftermath does not compel relinquishing the recognition that those compromised, unstable objects of modernity, ‘species’, have been lost along the way; but to suggest that these are fallible historical identifications of mutable lifeforms. This allows for a more capacious set of encounters, however strained, between the global biopolitics that make life continue, and the worlds of life’s aftermath. This version of life displaces the teleology of the Child and the Native with a disposition towards making life work among disturbed identifications (of species, of individual, and of boundaries between sacred and scientific environments). Offering prayers does not have to mean a nihilistic acceptance of rapidly disappearing forms. Ishiguro’s story acknowledges the difficulty of encountering life in the aftermath, sacred and scientific, finding something still moving in the encounters that would keep enhanced environments open to novel patterns of life.

## Data Availability

No data are available.

## Footnotes

Earlier published anthropological work focused on the Ainu alongside other Indigenous populations, their concepts of time, as comparative examples of shamanic practitioners, or their methods of healing ([Bibr R26][Bibr R27][Bibr R28]). These studies were not only significant in revising Japanese historical accounts of the Ainu, but also brought the Ainu into mainstream Anglophone academia as research subjects. Emiko Ohnuki-Tierney’s studies were published in the journals *American Ethnologist* and *Man*.

Takako Yamada carried out an earlier study on the Ainu classification of plants which was much more scientific in focus than the later work, focusing on their discrimination between plants, and their attention to ‘plant parts that are useful’ (1986, 141). This study, published in the *Japanese Journal of Ethnology*, includes a classification chart of Ainu plant taxonomy.
